# Myocardial first-pass perfusion imaging with hybrid-EPI: frequency-offsets and potential artefacts

**DOI:** 10.1186/1532-429X-14-44

**Published:** 2012-06-25

**Authors:** Pedro F Ferreira, Peter D Gatehouse, David N Firmin

**Affiliations:** 1 Faculty of Medicine, Imperial College, London, UK; 2 BRU, Royal Brompton Hospital, London, UK

## Abstract

**Background:**

First-pass myocardial perfusion is often imaged with a tailored hybrid centric interleaved echo-planar-imaging sequence, providing rapid image acquisition with good contrast enhancement. The centric interleaved phase-encode order minimises the effective time-of-echo but it is sensitive to frequency-offsets. This short article aims to show possible artefacts that might originate with this sequence, in the context of first-pass perfusion imaging, when frequency-offsets are present. Non-uniform magnitude modulation effects were also analysed.

**Methods:**

Numerical and phantom simulations were used to illustrate the effects of frequency-offsets and non-uniform magnitude modulation with this sequence in a typical perfusion protocol. *In vivo* data was post-processed to analyse the h-EPI’s sensitivity to the frequency-offsets.

**Results:**

The centric phase-order was shown to be highly sensitive to frequency-offsets due to its symmetrical phase slope. Resulting artefacts include blurring, and splitting of the image into two identical copies along the phase-encode direction. It was also shown that frequency-offsets can introduce signal loss and ghosting of the right ventricle signal into the myocardium. The *in vivo* results were confirmed by numerical and phantom simulations. Magnitude modulation effects were found to be small.

**Conclusions:**

Imaging first-pass myocardial perfusion with an hybrid centric echo-planar-imaging sequence can be corrupted with ghosting and splitting of the image due to frequency-offsets.

## Background

Myocardial first-pass perfusion imaging often uses an Echo-Planar-Imaging sequence in a multishot “hybrid” variety using a centric interleaved phase-encode order (“h-EPI”) [1-6] originally developed by Ding et al. [7]. This phase-encode order was tailored for first-pass myocardial perfusion, minimising the effective time-of-echo (TE) thus minimising susceptibility dephasing of signal within pixels, especially during first-pass of a strong paramagnetic contrast agent (CA), and minimising blood flow artefacts, while providing a good CA enhancement.

Interleaved h-EPI sequences offer rapid image acquisition, but are sensitive to frequency errors, which introduce phase evolutions throughout the echo-train. A technique known as echo time-shifting [8] is often used to smooth the phase evolutions along the phase-encode direction reducing potential frequency-offset artefacts. Although, even with echo-time-shifting conspicuous artefacts may still be generated for larger frequency-offsets [9]. The centric trajectory presented by Ding et al. [7] may disrupt this smooth-phase evolution by introducing a phase discontinuity at the centre of k-space for regions with frequency-offsets, which may translate into image artefacts.

In this work we examined potential artefacts that frequency-offsets may introduce in a first-pass perfusion imaging study setup with a centric interleaved h-EPI sequence; specifically whether frequency-offsets could cause substantial image degradation and subendocardial dark rim artefacts (DRA) mimicking real perfusion defects *in vivo* and hindering diagnostic quality. Non-uniform magnitude modulation effects were also analysed, specifically if at peak CA concentration, i.e. at short T1 and T2*, the signal modulation with the centric phase-order creates additional artefacts.

Myocardial perfusion imaging is routinely performed with this sequence on 1.5 T Siemens Avanto scanners in our department. Frequency offsets and the artefact in the left-ventricle are controlled by mandatory frequency adjustment in a typically 130×130×130 mm volume placed on the left heart prior to perfusion imaging. The main field shimming is set to the service engineering settings (“tune up” mode) with no *in vivo* shimming adjustments. Provided these instructions are followed correctly, the off-resonance artefact does not occur in the left ventricle; otherwise it would be likely to affect patients erratically with potentially severe consequences depending on the scanner frequency used (as illustrated below). We believe it is therefore important to report this artefact along with its routine elimination by frequency adjustment performed locally in the left ventricle.

## Methods

In order to study the effects of frequency-offsets on a centric interleaved h-EPI sequence, simulations were performed both numerically by using MATLAB (Mathworks, Natick, USA-MA), and on simple phantom models of first-pass perfusion. This work followed after *in vivo* data drew our attention to the problem. *In vivo* data was visually assessed and its raw-data modified in MATLAB by artificially introducing different frequency-offsets.

### Numerical and phantom simulations

MATLAB was used to calculate the phase evolution in k-space produced by different frequency-offsets. The simulated protocol was similar to the protocol routinely used in our centre for first-pass perfusion: h-EPI sequence with an echo-train of 4 echoes; TE (time of echo (first echo)) 1.12 ms; TR (time of repetition) 5.6 ms; TI (time of inversion) 100 ms; flip angle 28°; voxel size 2.3×2.3×8 mm, readout/phase FOV (field of view) 370/300 mm, matrix size 160×128 pixels, bandwidth 1735 Hz/pixel; TGRAPPA [10,11] R = 2; echo-spacing 0.9 ms, time per slice 140 ms (including saturation recovery). The simulated sequence had a centric interleaved phase order and echo-time-shifting. The phase evolutions were computed by assuming a zero phase at the centre of k-space. The phase obtained at any k-space point was calculated for a range of different frequency-offsets from 0 to 250 Hz (approximately 4 ppm (parts per million) at 1.5 T). The PSF (point spread function), i.e. the inverse Fourier transform of the phase evolution over the 2D k-space and its convolution with an image consisting of a homogeneous disc (diameter 80 mm) were then computed for each offset.

The effects of non-uniform magnitude modulation during data acquisition were also numerically simulated for three pairs of (T1, T2*) values in milliseconds: (120, 20), (13, 5), and (5, 2). The T2* values were estimated as approximately half of the T2, based on first-pass measurements in the left ventricle [12]. The T1 and T2 relaxation times correspond to CA concentrations in the RV of ≈1.6, 16 and 46 mmol/L, using relaxivities *r*_1_=4.5, *r*_2_=5.1 L mmol^−1^ sec^−1^. The peak concentration of CA in the RV was estimated at 11.7±2.3 mmol/L [13] for 0.1 mmol/kg of 500 mmol/L Gd-DTPA injected at 4 mL/s with 20 mL saline flush. However, the RV peak concentration in Ishida et al. was an average over 10 subjects, and a combination of inter-subject variability, stress imaging and higher injection concentration could lead to much higher peak RV concentrations. Considering these (T1, T2*) values and the hybrid-EPI protocol defined above, the transverse signal at the TE of each acquired phase-encode line was calculated and its modulation effect in the k-space of a homogeneous disc was computed.

Frequency-offsets in the range of 0 to 250 Hz were also simulated in a phantom. This phantom consisted of a circular cylindrical container (diameter 80 mm) with a solution of Gd-DTPA, at a concentration of 12.5 mmol/L, forming an approximate relaxation-time model of the LV during CA first-pass. The phantom was imaged with the cylinder long-axis along B0, and the image plane was perpendicular to the long-axis, resulting in a disc similar to the numerical simulation. At the time of the scan, the resonance frequency was carefully calculated for the solution of Gd; a 3D box containing only the phantom was defined by the user in the FOV, this volume was used by the scanner to calculate the average resonance frequency. This frequency was then manually overridden with different offsets before running an h-EPI sequence with a protocol identical to the one used in the numerical simulations.

A second phantom consisting of a hollow diamagnetic cylinder made with undiluted gelatine (Hartley’s strawberry jelly, Spalding, UK) in the same container as the previous phantom was also imaged. Just before scan time, the phantom was removed from a fridge and the hollow cylinder was filled with cold water doped with 12.5 mmol/L Gd-DTPA, simulating once again the blood pool in the left ventricle during the peak first-pass of CA (contrast agent). This phantom presents a model of the border between the myocardial wall and the LV, aiming for similar T1, T2, and susceptibility as the LV and myocardium during the CA first-pass (jelly T1 = 74 ms, T2 = 12 ms). Short-axis (SA) images of this phantom (i.e. perpendicular to the cylinder long axis) should therefore have similar blood/myocardium contrast and approximate susceptibility effects as the heart. This phantom was also imaged with a similar h-EPI protocol at two different resonance frequencies; one approximating the average frequency of the whole of the phantom, and a second approximating the average frequency for the Gd-solution only. All phantom work was performed at 1.5 T.

#### In vivo

All *in vivo* results shown in this article were also collected at 1.5 T (Avanto, Siemens; Erlangen, Germany), with a centric interleaved h-EPI sequence [7]. All patients had signed permission for their images to be used in ethically approved clinical research.

The imaging protocol was similar to the one used in the numerical and phantom simulations, using posterior/anterior phased array cardiac coils. Perfusion imaging was performed in three short-axis slices (basal, mid and apical) for each heartbeat for at least 50 R-R intervals at stress and then repeated at rest. Maximal hyperaemia was induced by a continuous intravenous infusion of adenosine at a rate of 140 *μ*g/kg/min for at least 4 minutes prior to imaging. At the time of scan, patients were injected with Gadobutrol (Gd-DO3A-butriol) (Gadovist-Bayer, Germany) with a concentration of 1.0 mol/L. A dose of 0.1 mmol/kg of body weight was injected at a rate of 3.5 mL/s followed by 25 mL of a saline flush at 7 mL/s.

For two *in vivo* perfusion studies, accumulated phases corresponding to a range of scanner reference frequency-offsets were added to the stored raw-data and images were repeat-reconstructed in the scanner to examine h-EPI’s sensitivity to the frequency-offsets. The image reconstruction was identical to that applied to the original data. The processing of the raw-data was performed in a standalone PC with MATLAB.

## Results

### Numerical simulations

**Figure 1:**
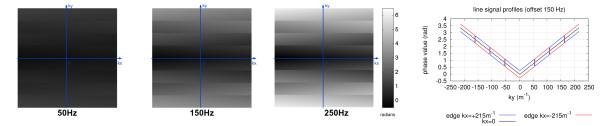
**Phase evolution.** Left: Phase (radians) over k-space simulated on MATLAB for three different frequency-offsets (50 Hz, 150 Hz, and 250 Hz). The phases were calculated considering 0 radians at the centre of k-space and echo-time shifting. Right: Line signal profiles along the phase-encode direction for the 150 Hz offset. It is shown three line profiles, two for the two frequency-encode edges of k-space (red: *kx*=−215m^−1^, blue: *kx*= + 215m^−1^) and one for the centre (*kx*=0m^−1^).

Figure 1 shows the phase evolution map (in radians) of the k-space for three different frequency-offsets (50, 150, and 250 Hz). As expected, the phase evolution maps scale linearly with the frequency-offset. For an offset of 250 Hz there is a phase range of more than 6 radians. The frequency-offsets with echo-time-shifting produce a smooth phase slope along the phase-encode direction for values of zero in the frequency-encode only. Phase discontinuities exist for all other frequency-encode values. Because the k-space trajectory simulated is a centric interleaved, the phase-slope inverts across the centre of *ky*.

**Figure 2:**
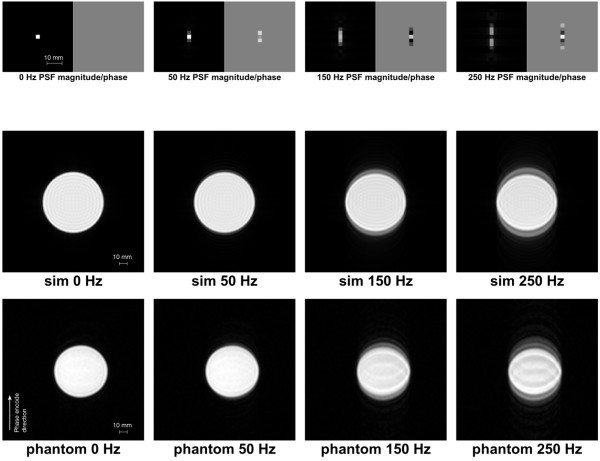
**PSF, numerical, and phantom simulations.** Top row: PSF magnitude and phase for four different frequency-offsets: 0, 50, 150, 250 Hz. The phase images were thresholded with the magnitude image, the phase values were nulled for the corresponding magnitude points with <15*%*of the peak magnitude. The images are zoomed in to the centre, original *FOV*=370mm. Middle row: The effects of those frequency-offsets on a numerical simulated homogeneous disc. Bottom row: Phantom results for the same frequency-offsets.

The resulting PSFs in the image space were calculated from the phase over k-space, enabling a better understanding of the effects of a frequency-offset in an object. The PSF’s magnitude and phase images are shown in Figure 2 (top row) for the same three frequency-offsets.

The PSF images show that the frequency-offsets will result in blurring and localised paired ghosting of the image mainly along the phase-encode direction, accompanied by phase variations. These effects were simulated for a homogeneous disc as shown in Figure 2 (middle row). The frequency-offsets caused blurring and splitting of the image into two superimposed copies shifted in opposite direction along the phase-encode direction as expected. The distance between these two copies increases with the offset.

**Figure 3:**
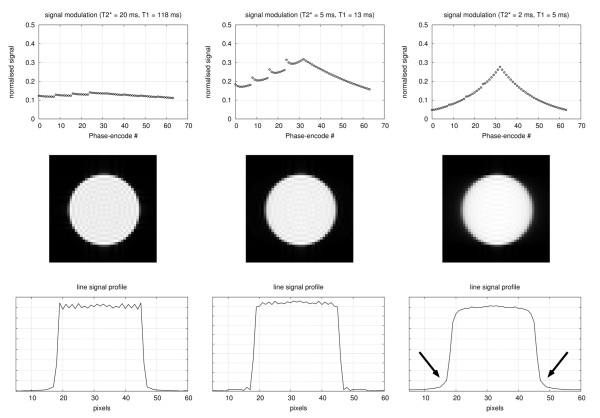
**Non-uniform magnitude.** Non-uniform magnitude modulation during data acquisition for (T1, T2*) values of (120, 20); (13, 5); and (5, 2) ms. Top row: normalised signal for each phase-encode line acquired with a centric interleaved order (parallel imaging with R=2). Middle row: the magnitude modulation effects simulated on a homogenous disk. Bottom row: line signal profile across the centre of the disk along the phase-encode direction. The arrows point to the blurred edge of the disk.

The effects of non-uniform magnitude modulation are shown in Figure 3. As the (T1, T2*) values decrease the magnitude modulation gives rise to blurring (arrows), although this effect is only noticeable at very low values of T2*.

### Phantom simulations

Similar frequency-offset results were obtained for the cylindrical phantom with the solution of Gd. Figure 2 (bottom row) shows the phantom images at the same frequency-offsets as shown for the numerical simulations. The results show similar ghosting and blurring along the phase-encode direction.

**Figure 4:**
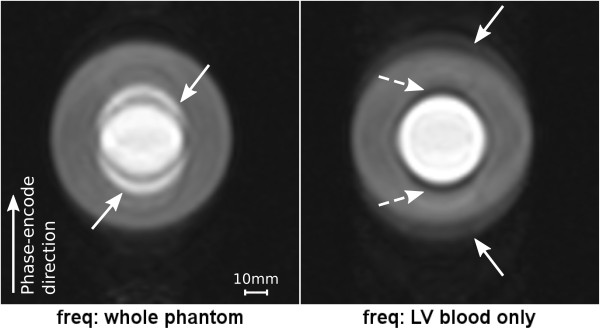
**Hollow diamagnetic cylinder phantom.** Gelatine phantom with the solution of Gd. The images show two h-EPI scans with two different frequency adjustment volumes: Left: entire phantom volume; Right: Gd-solution volume only.

The results for the phantom with the hollow diamagnetic gelatine cylinder filled with a solution of Gd, simulating a short-axis image of the heart during CA arrival in the LV are shown in Figure 4. When the reference frequency was set to the entire phantom volume (left), the LV blood “split” into two superimposed copies ± 5 mm aside its true location (arrows) along the phase-encode direction. Conversely using the blood frequency (right), the LV blood was imaged sharply, whereas ghosting can be seen for the off-resonant “myocardium” (solid arrows); a dark rim can also be seen in the “endocardial” border (dashed arrows). The difference in centre frequency between the two images was 181 Hz.

#### In vivo

**Figure 5:**
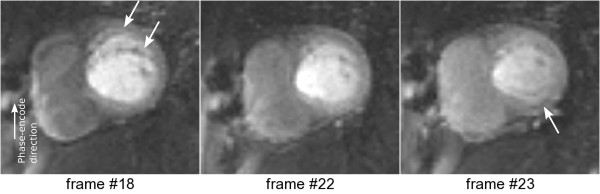
**First pass perfusion example.***In vivo* example with severe frequency-offset artefacts. Three frames are shown during CA first-pass. A split of the anterior wall is visible along the phase encode direction (arrows in frame #18). A dark rim artefact is also visible in frame #23 (arrow).

Clinical *in vivo* examples of this effect were found. Figure 5 shows an extreme example of frequency-offset artefacts visible during first-pass perfusion, manifesting as ghosting along the phase-encode direction. Please note that such extreme artefacts are rare and possibly due to an erroneous frequency adjustment before the scan. Unfortunately the scanner’s raw-data was not stored for this particular study.

**Figure 6:**
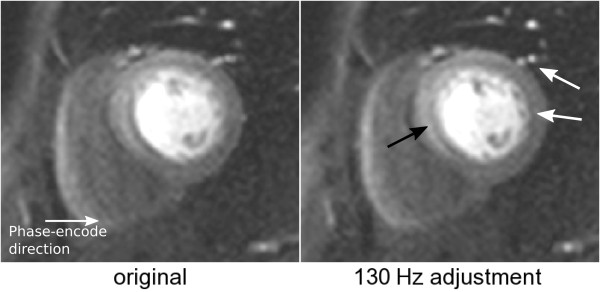
**Adjusted*in vivo*data.** Left: original perfusion image. Right: original image after a +130 Hz adjustment. The black arrow points to the blurred septal wall, while the white arrows point to the sharper lateral wall and blood vessels.

A less conspicuous artefact is more commonly found in perfusion studies and an example is shown in Figure 6. In this particular case, the original data drew our attention due to the blurred lateral wall. The raw-data was processed in MATLAB and several different frequency-offsets in relation to the original data were added. It can be seen that with 130 Hz adjustment, the region proximal to the lateral wall becomes sharper (white arrows), but at the same time degraded the septum (black arrow), suggesting a frequency slope across the heart. A clue that the sharpness increased for the lateral wall is the increase in Gibbs ringing, with a reciprocal effect for the septal wall.

**Figure 7:**
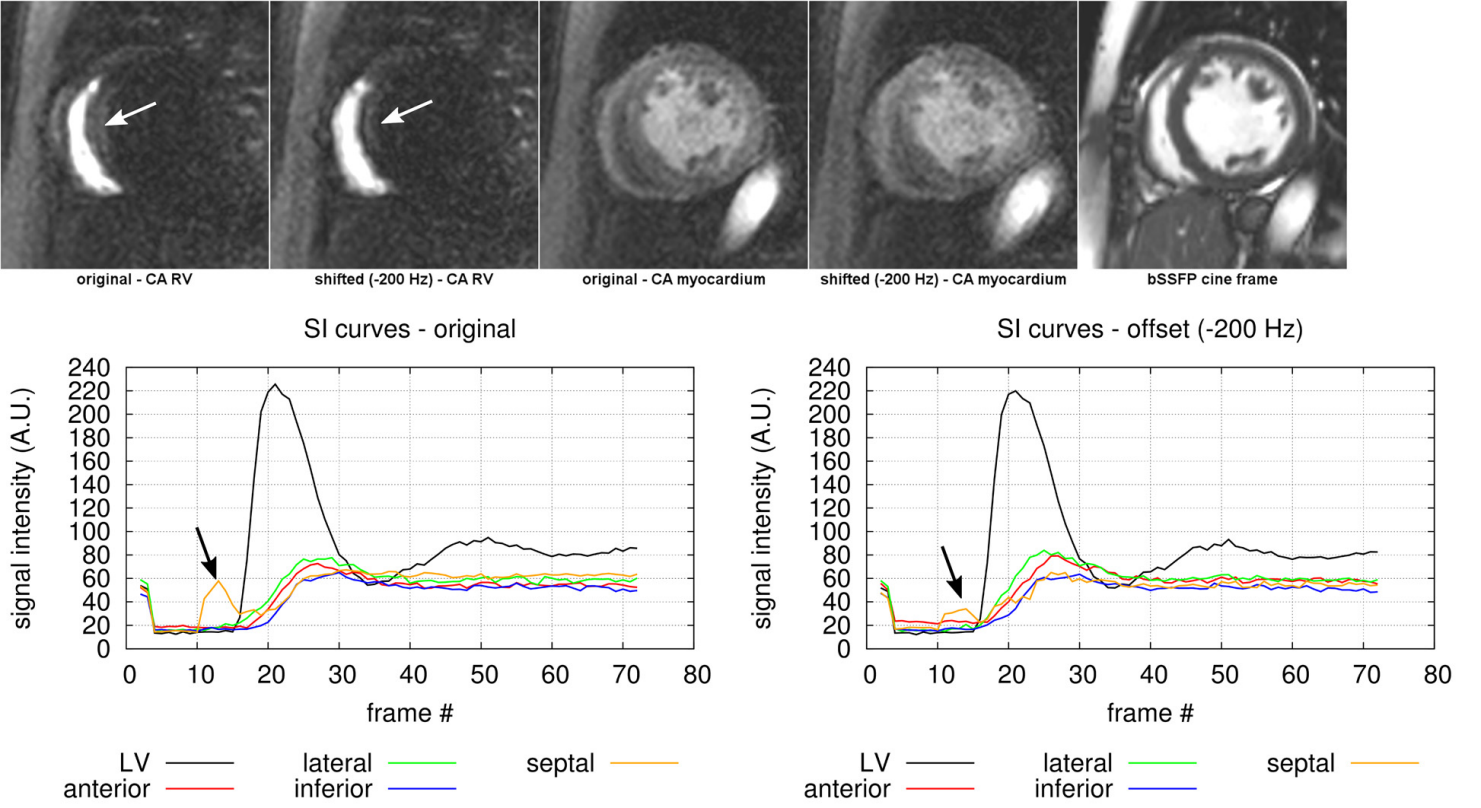
**Right ventricular ghosting.** Top from left to right: The original basal SA image at CA arrival in the RV, the corresponding frame after an adjustment by −200 Hz (the white arrows show the RV ghosting). The original basal SA image during myocardial perfusion and the correspondent frame after the same −200 Hz adjustment. Finally a SA basal slice of a bSSFP cine at a similar slice position and at a similar heart phase as the perfusion data for morphological reference. Bottom: The average SI curves for the septal, anterior, lateral and inferior myocardial wall segments, and for the LV blood pool. On the left is shown the original data, and on the right the frequency adjusted (−200 Hz) data.

One other artefact commonly seen is ghosting of the RV blood signal in the septal segment of the myocardium as shown in Figure 7. This artefact can easily be shown in the myocardial SI (signal intensity) curves in the earlier stages of a perfusion scan. When the CA arrives in the RV the bright signal spreads along the phase encode direction, which often affects the septal region of the myocardium. After an adjustment of the resonance frequency in the raw-data by −200 Hz, the RV ghosting in the septal region is reduced (white arrows on the SA magnitude images); this can also be seen in the plots of the SI curves for the septal segment (black arrows). Even though this frequency adjustment reduced the RV ghosting, it also worsened the sharpness of the myocardial wall, as can be seen in the SA magnitude images during myocardial perfusion acquired approximately 15 heart-beats later on.

The magnitude of the ghost measured in the septum is only approximately 25% of the RV brightness which is smaller than the 50% that would be expected if caused entirely by off resonance splitting of the PSF. The reason for this is possibly complex field distortions in this region and not an homogeneous offset in the entire RV blood pool. The non-uniform magnitude modulation simulations in Figure 3 do not suggest that this is a major factor either.

## Discussion

This work has shown that the frequency-offsets, typically encountered in a first-pass myocardial perfusion scan, can generate conspicuous artefacts when using an h-EPI sequence with an interleaved centric phase-order. This article follows from Mugler’s et al. [9] work, where it is shown ghosting and blurring artefacts for linear interleaved h-EPI sequences. Here we analyse a centric phase-order commonly used in myocardial perfusion imaging which is more sensitive to off-resonance spins by adding a symmetrical phase slope resulting in a broadening of the PSF along the phase-encode direction, eventually splitting into two identical copies of the imaged object. Phase oscillations in the PSF can also cause signal loss, which can possibly create DRAs. These results were confirmed by numerical and phantom simulations with good agreement.

The centric phase-encode order was tailored for first-pass myocardial perfusion because it minimises the effective TE [7]. A non-centric (linear) interleaved trajectory would have a phase slope half as steep, and more importantly it would not change direction in the centre of k-space, therefore the PSF would not split in two and would shift only half the distance along one phase-encode direction. However it would introduce more flow artefacts and signal loss due to the longer effective TE.

Non-uniform magnitude modulation response during data sampling can result in additional blurring. This effect is small when considering typical concentrations of CA found in the heart [12,13], except possibly during the first-pass of Gd in the right ventricle due to its very short T2* at peak concentration. The modulation shape is approximately symmetric at the centre of k-space due to the centric phase order, avoiding edge enhancement artefacts.

Off-resonance is typically produced by B0 inhomogeneity or changes in magnetic susceptibility. Reeder et al. [14] measured a 70-100 Hz difference between the myocardium and the posterior vein region for a short-axis slice without any contrast agent. Shah et al. [15] measured offsets of up to 90 Hz along the heart. Additionally, frequency-offsets are also produced during the first pass of a strong paramagnetic Gd based CA. In a previous publication our group measured *in vivo* at rest an additional offset of up to 130 Hz between the LV blood pool and the myocardium during the peak concentration of Gd in the LV [16]. The net effect of these offsets is likely to cause noticeable artefacts, including blurring and ghosting.

An improved cardiac shim [17] will minimise local frequency-offsets, and provide a more accurate fat suppression, a requirement of EPI sequences, although usually fat suppression is not effective in the entire FOV. The off-resonance of fat is approximately 210 Hz at 1.5 T, therefore incomplete suppression of fat will results in its signal being split. This can potentially affect myocardial signal for nearby fat such as epicardial fat.

Also as shown by Mugler et al. [9], the phase discontinuities increase away from *kx* = 0, increasing artefacts in sharper edges such as the subendocardial border due to its sharp discontinuity in signal between the bright LV blood pool and the myocardium. Unfortunately this is where the early perfusion defects occur in coronary artery disease. These artefacts can therefore potentially obscure a thin subendocardial perfusion defect.

Another shown difficulty arising from frequency-offsets is the ghosting of the RV signal in the myocardium at the earlier stages of the perfusion study. Although this artefact peaks before LV first pass, it will complicate any analysis dependent on myocardial SI curves, such as determining myocardial signal upslope or fully quantitative blood flow analysis.

The distance between the two identical copies of the imaged object can be obtained from the Fourier Transform Shift Theorem. The following equations consider the complete raw-data after computation of the missing phase-encode lines by parallel imaging. The parallel imaging algorithm introduces intermediate phase-encode lines but does not change the resulting phase slope over ky in radians per inverse meter. If echo-time-shifting is included, the phase difference *θ* between the centre and each edge of ky is given by *θ*=$2\Pi \times \mathrm{\Delta f}\times \left(\mathrm{\Delta T}\times \left(n_{\text{echoes}}-1\right)+\frac{\mathrm{\Delta T}}{n_{\text{shots}/2}}\times \left(n_{\text{shots}/2}-1\right)\right)$, where *Δf* is the frequency offset, *ΔT* is the time interval between echoes of the EPI readout, *n*_echoes_ is the number of echoes per EPI readout, and *n*_shots/2_is the number of excitations needed to completely fill half the k-space. According to the Shift Theorem this will result in a shift of $2\times \mathrm{\Delta f}\times \left(\mathrm{\Delta T}\times \left(n_{\text{echoes}}-1\right)+\frac{\mathrm{\Delta T}}{n_{\text{shots}/2}}\times \left(n_{\text{shots}/2}-1\right)\right)$ pixels for this k-space half. Considering a symmetric phase slope in the other half of k-space, the resulting distance between the two copies is twice this distance along the phase encode direction. In mm the distance between the two copies is therefore:

(1) $$\;\begin{array}{cc} d & =4\times \mathrm{\Delta f}\times \left(\mathrm{\Delta T}\times \left(n_{\text{echoes}}-1\right)+\frac{\mathrm{\Delta T}}{n_{\text{shots}/2}}\right)\\ \;\times \left(\left(n_{\text{shots}/2}-1\right)\;\right)\times \frac{\mathrm{FO}V_{\text{pe}}}{N_{\text{pe}}} \end{array}$$

where *FOV*_pe_ is the FOV in the phase-encode direction, and *N*_pe_ is the total number of phase-encode lines after parallel imaging computation. It is emphasised that all of the parameters in these equations are considered for the complete raw-data set after calculation of the missing phase-encode lines by parallel imaging. The predictions from these equations are in agreement with the numerical and phantom results.

As an example for an offset of 210 Hz as encountered in fat, the distance between the two copies is approximately 6.9 mm considering the protocol described in the Methods section (*ΔT*=0.9 ms, *n*_echoes_=4, *n*_shots/2_=8, *FOV*_pe_=300 mm, *N*_pe_=128 px). The distance increases with the number of echoes per EPI readout; therefore in order to minimise artefacts they must be kept to a minimum, which will increase the time of image acquisition presenting a trade-off between frequency-offset and motion artefacts. It must be pointed out that the distance does not directly depend on the use of parallel imaging.

Some MR scanners provide local resonance frequency adjustments as performed in these experiments, minimising possible frequency-offsets and suppressing epicardial fat more reliably, although this adjustment is only approximate because is performed prior to the injection of Gd, which will inevitably modify B0 in the heart during first-pass. B0 distortion during first-pass is not only time variant, but also spatially dependent, with different frequency-offsets for the LV and myocardium; even within the myocardium there are positive frequency-offsets along the in-plane component of B0 and negative frequency-offsets across the in-plane component of B0 [16]. Therefore frequency-offsets will be unavoidable during first pass and likely to peak during peak concentration of Gd in the LV, unfortunately a key timing to detect real perfusion defects.

Reducing CA dose will potentially reduce frequency-offsets during first-pass, but it will also reduce CNR and hinder diagnostic confidence.

All the work shown here was done on a 1.5 T scanner. A higher field scanner will present even more challenges due to the higher frequencies.

## Conclusions

We have shown that blurring and splitting of the image along the phase-encode direction can occur when imaging myocardial perfusion with a centric h-EPI.

These frequency-offsets vary locally over the heart. Careful adjustments to the resonance frequency and cardiac shimming are essential to minimise these artefacts.

## Competing interests

The authors declare that they have no competing interests.

## Authors’ contributions

All authors contributed in the design, and intellectual conception. PF, PG, and DF participated in revision, analysis and interpretation of the data. All authors read and approved the final manuscript.
